# A Calibration and Quality Assurance Program for Environmental Radon Measurements

**DOI:** 10.6028/jres.095.013

**Published:** 1990

**Authors:** Isabel M. Fisenne, Andreas C. George, Helen W. Keller

**Affiliations:** Environmental Measurements Laboratory, U.S. Department of Energy New York, NY 10014

**Keywords:** calibration, fast pulse ionization chambers, quality assurance, radon, radon calibration facility

## Abstract

The ideal facility for assessing the quality of radon measurements at environmental levels consists of: (1) an instrument whose response to radon and its progeny is determined from measurements of a certified or standard ^226^Ra source, and (2) a calibration room with a known radon concentration.

The linkage between these two elements and additional quality control requirements are discussed here for some Environmental Measurements Laboratory radon measurements programs.

## 1. Introduction

The Environmental Measurements Laboratory (EML) of the U.S. Department of Energy (DoE) has considerable experience in the measurement of environmental levels of ^222^Rn. The mainstay of radon measurement programs at EML has been fast pulse ionization chambers. Originally designed and constructed in 1948 by the Health and Safety Division of the U.S. Atomic Energy Commission, New York Operations Office, the chamber systems incorporated a number of innovations which greatly simplified and improved the measurement of radon in breath and air samples.

The original set of four pulse ionization chambers has increased to nine over the intervening 40 years. The development of a reliable and flexible radon calibration chamber at EML completed the requirements for a facility to assess the quality of environmental radon measurements.

## 2. The EML Pulse Ionization Chamber Systems

An extensive report [1] has been published which describes the design of the EML fast pulse ionization chamber systems, the methods of calibrating these chambers and the programs dependent on measurements performed in these chambers. The unique features of the chamber systems will be briefly reviewed in this report.

An EML cylindrical stainless steel ionization chamber has a volume of 1.78 L and a nominal total volume of 2 L, including the purification system. Each chamber is constructed with a plug in the baseplate to accommodate an electrodeposited standard for determination of the alpha counting plateau. The purification system for the removal of oxygen and water vapor from air and breath samples has three advantages over other fast pulse detection systems: simplicity; a small dead space to chamber volume ratio; and dichotomy, i.e., simultaneous access to one or two ionization chamber.

To perform a radon measurement, the air or breath sample container is overpressured with hydrogen gas. The sample is transferred to the evacuated system in which the gas flow rate is regulated by capillary orifices. The oxygen in the sample and the added excess hydrogen combine to form water after passing through cartridges containing platinum black catalyst. The water vapor is removed with a calcium chloride column. The transfer of the sample to an ionization chamber is completed by the flow of forming gas (85% nitrogen, 15% hydrogen) through the sample vessel and purification system into the chamber. The chamber is pressurized to 35 kPa gauge, and then sealed and the sample is measured for a minimum of 17 h.

### 2.1 Variables in the Calibration of the Pulse Ionization Chamber Systems

The EML pulse ionization chamber systems have been calibrated with several different series of National Institute of Standards and Technology (NIST) Standard Reference Material (SRM) ^226^Ra solutions by the radon emanation method since 1961. During the ensuing 28 years, a number of variables have been checked empirically to determine any effect on the calibration factor.

Two types of bubblers or gas washing towers have been used and both have been tested to determine the transfer efficiency from solution to the chambers, the possible effect of acidity or alkalinity on the gas transfer, the rate of loss of radon from the bubblers, and the quantity of ^226^Ra in the bubbler. A significant finding was that the addition of a few drops of wetting agent such as aerosol OT to a radium-bearing acid solution in the bubbler and refrigeration of the bubbler prior to emanation yielded an efficient and reproducible transfer of radon (see reference [1]).

The effect of pressure within the pulse ionization chamber on the calibration factor was checked by measuring the radon emanated from a solution at both atmospheric and 35 kPa gauge pressure. No difference was found in the calibration factor.

The effect of the gas mixture in the ionization chamber was checked to determine whether this would affect the calibration factor. First a 0.16 L room air sample to which hydrogen had been added was transferred to the chamber, then a ^226^Ra standard solution was emanated into the same chamber. No difference was found in the calibration factor from that determined by emanating radon from a ^226^Ra standard solution with forming gas.

To determine whether substantial shifts occurred because of the gas filling, tests were made using the pulse size spectra of the forming gas background counts, an emanated NIST SRM ^226^Ra solution and air samples. Only one ionization chamber was used in this series of measurements. For this chamber, the tripping level, that is, the pulse size expressed in volts below which pulses are not recorded, was 0.8 V. For the forming gas background measurements, 20% of the pulses were below 3.0 V with the median pulse size of 6.4 V and a maximum of 10.3 V. The NIST emanated ^226^Ra solution spectrum showed 20% of the pulses below 4.2 V with a median pulse size of 6.4 V and a maximum of 10.3 V. Two 0.08-L air samples were collected in the EML radon calibration chamber and measured on consecutive days. The first sample was transferred to the ionization chamber and measured at atmospheric pressure. The second sample was transferred to the ionization chamber and measured at 35-kPa gauge. The sample spectra were identical and showed that 20% of the pulses were below 4.8 V, the median pulse size was 6.0 V and the maximum was 9.5 V. Lastly, a 0.16-L ^220^Rn (thoron) sample was collected in the EML radon calibration chamber and measured in the ionization chamber at a pressure of 35 kPa gauge. The thoron sample spectrum showed that 20% of the pulses were below 4.0 V, the median pulse size was 6.4 V and the maximum was 10.3 V.

It was concluded from these series of measurements that the ionization chambers are insensitive to the gas mixture as long as oxygen and water vapor are removed and also that there is no difference in the calibration factor for the chambers determined at atmospheric or 35 kPa gauge.

## 3. Summary of NIST SRM ^226^Ra Solution Certification

The calibration factors for the EML pulse ionization chambers are determined from measurements of NIST SRM ^226^Ra solutions. Because the estimation of the systematic error for measurements in the ionization chambers includes the total uncertainty ascribed by NIST to SRM ^226^Ra solutions, it is important to review the practices used in the certification process. Table 1 summarizes the evolution of the NIST certification process from 1940 through 1984. Over this period, significant changes have been made in the choice of the primary instrument used in the NIST measurements and in the way in which the ^226^Ra value is certified. Except for the 1940 series, the information in the table was taken directly from certificates of ^226^Ra solution used to calibrate the EML pulse ionization chambers. Historically, the ^226^Ra solutions have been certified in mass units. Until 1978, the certification was made on a mass per ampoule basis. Since that time the certification has been given as a concentration, but still as mass of ^226^Ra g^−1^ of solution. However, a suggested half-life for ^226^Ra did not appear on the certificate until 1984.

The 1940 and 1947 ^226^Ra series were certified on the basis of comparative measurements against the national primary radium standard using a gold-leaf electroscope [2]. The total uncertainty for these measurements was less than 1%. In 1957, the primary instrument for the ^226^Ra measurements was the radiation balance [3] with the electroscopic measurements used for confirmation purposes. The standardization was by direct comparison with the national primary radium standards. A reevaluation of this series a decade later resulted in an increased uncertainty. The 1965 series “was calibrated by comparing its *γ* ray emission rate with those of a series of standards prepared for material that was compared, in the NIST radiation balance, with the national radium standards. The *γ*-ray emission rates were compared in the NIST 4 *π-γ* ionization chamber.” The estimated overall uncertainty was given as 0.50%. The 1967 series was calibrated by comparison with the 1957 series in the radiation balance and 4 *π-γ* ionization chamber. The uncertainty was quoted as 3.6%. The 1978 series marked the first time that ^226^Ra was certified on a concentration basis, that is, the mass of ^226^Ra g^−1^ of solution. Once again the material was certified by comparison of its *γ*-ray emission rate with the 1957 series of standards. Only the 4 *π-γ* ionization chamber was used in this certification process. The estimated total uncertainty for this series was 1.34% for the concentration value and 1.53% for the ampoule value. The series was certified from measurements made in the pressurized 4 *π*-*γ* ionization chamber calibrated with the national radium standards. The uncertainty in the ^226^Ra concentration was assessed as 1.30%.

The total uncertainty in NIST SRM ^226^Ra solution values has generally been on the order of 1 %, but it is only one of several possible systematic errors which must be considered in the estimation of total uncertainty by an SRM user.

## 4. EML Pulse lonization Chamber Calibration Factors and Estimated Total Uncertainty in Radon Measurements

The EML pulse ionization chambers have been rigorously calibrated five times between 1960 and 1984 using NIST SRM ^226^Ra solutions. In each case, the SRM ^226^Ra was from a different series. The determination of the calibration factors for the ionization chambers requires 2 to 3 months. The 1984 calibration of the ionization chambers will be used to illustrate the process at EML. The details of previous calibrations of the chambers are documented in reference [1].

When the 1984 NIST SRM ^226^Ra series became available, EML purchased several ampoules. One ampoule of SRM 4950 E having a ^226^Ra concentration of 7.566×10^−11^ g per g of solution was selected for the program. Nine emanation flasks or bubblers, which had been checked and found to be free of contamination, were needed for the measurements. A measured amount of l*N*HCl was added to each flask. The NIST SRM ^226^Ra solution was transferred to a plastic ampoule used for weighing and delivery of solutions. A weighed aliquot of the ^226^Ra solution was delivered to each of the nine bubblers. The bubblers were de-emanated with forming gas to establish time=0 for the build up of radon. Over the course of the program build up periods ranged from 2 to 10 d. The measurement protocol was devised as a 9×9 matrix, that is, the radon from each of the nine bubblers is emanated once into each of the nine chambers. This allows us to detect any bias in a particular bubbler or chamber. The redetermined calibration factor for the chambers was calculated from the 81 measurements. Only a single calibration factor need be used for the nine chambers since the mean value obtained for each chamber agrees with the remaining eight chambers within the standard deviation of the measurements. The 81 measurements have been shown to be normally distributed.

Table 2 summarizes the results of the major calibrations of the EML ionization chambers and gives an estimation of the magnitudes of the random and systematic errors associated with the measurements. The EML total uncertainty is the linear sum of the errors.

## 5. Quality Control and Quality Assurance Program for the EML Pulse lonization Chambers

As with any instrument, routine checks and maintenance are required for proper performance of the pulse ionization chambers. The background count rate of each chamber is measured with forming gas every weekend and occasionally during the work week to ensure against temporal bias. The background count rates for the nine chambers in service at present range for 12 to 20 counts h^−1^. A control chart of the weekend background count rates is maintained for each chamber. At the beginning of the work week, the average background count rates for the current year and the running average including the results from previous years are calculated for each chamber. The weekend background information is used to initiate corrective actions if deemed necessary. Over a period of years, the background count rate of a chamber increases due to the build up of long-lived radon progeny on the interior surfaces. The increase is a direct result of exposure in terms of Bq h^−1^. The background count rate may be reduced by dismantling and electropolishing the chamber. This procedure requires a lengthy recalibration of each chamber and is therefore undertaken only after a period of several years.

Occasionally the ionization chambers systems are checked for electrically generated noise by filling the chambers with room air. The oxygen in the air effectively reduces the pulse size below the tripping level of the electronics and only electrically generated pulses are registered during the overnight measurement period. The electronic “noise” in the chamber systems is less than 1 count in 4 h.

The platinum black catalyst and calcium chloride columns are kept free of water vapor by maintaining these cartridges imder vacuum except during sample introduction.

The calibration factor for the chambers is checked at least quarterly by emanating radon from a standard radium bubbler. It is important to emphasize that a bubbler containing radium for calibration purposes is only used for 1 year at EML.

EML participates in the Environmental Protection Agency Water Cross-Check Program for ^226^Ra analyses. The results over the last 4 years show agreement within ±5% of the expected value. EML also administers the Quality Assessment Program for the DOE Office of Environment, Safety and Health. The ^226^Ra analyses for soil, vegetation, and tissue are performed by radon emanation and the results are compared with those of DOE contractor laboratories. When possible, EML participates in the development of consensus standards for ^226^Ra in natural matrix standards. These exercises are usually initiated by organizations such as the International Atomic Energy Agency and NIST.

Quality assurance for measurements of radon in air samples is provided through a series of national and international intercomparison exercises in which EML is both a sponsor and a participant. The facility used in these exercises is described in the next section.

## 6. The EML Radon, Thoron, and Progeny Calibration Facility

The EML radon, thoron, and progeny calibration facility was constructed to provide a range of well-controlled environmental conditions. Experiments are conducted in this calibration facility to assess the accuracy of the methods and instruments used to measure radon and thoron and their progeny in occupational and non-occupational settings. Research studies are conducted on the environmental factors which affect the physical behavior of the progeny.

The calibration facility consists of seven principal components:

1. radon and thoron source generation systems,
2. anteroom to the exposure chamber,
3. exposure chamber,
4. environmental chamber and air flow control system,
5. radon, thoron, and progeny monitoring instruments,
6. aerosol generating system,
7. environmental control panel and data acquisition system.

For the purpose of this paper our discussion will be restricted to the generation and measurement of a controlled radon atmosphere.

### 6.1 The Radon Calibration Chamber

#### 6.6.1 The Physical Layout of the Exposure Chamber

**Figure 1:**
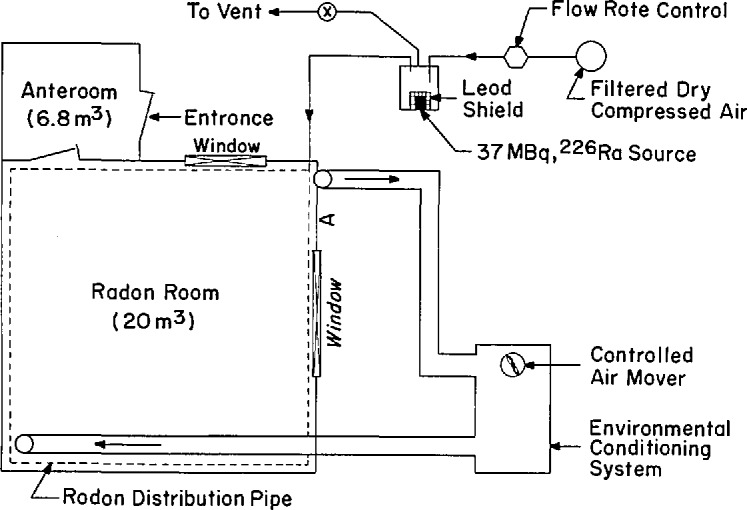
Schematic diagram of EML’s radon calibration chamber.

The EML radon calibration chamber is a 2.82×2.82×2.4 m aluminum clad chamber with a separated anteroom entering into the main room. The volume of the main room is 19.4 m^3^ Entry to the exposure room through the double door system in the anteroom serves to minimize the influx of adjacent room air into the main room. There are six access ports located on one wall of the exposure room through which small instruments can be introduced or samples can be taken without entering the exposure room. The exposure room is equipped with multiple electrical outlets, fluorescent lighting and a two-way communications system. The exposure room is viewed from the outside through two large glass windows. A schematic diagram of the essential features of the calibration room is shown in figure 1.

#### 6.1.2 Radon Delivery System

The radon source is a 37 MBq (1 mCi) dry radium bromide source housed in a shielded 0.2 m^3^ metal drum. The drum and its three input/output ports are hermetically sealed. Compressed air is filtered and dried prior to introduction into the drum. The air flow through the drum is maintained at a constant rate of 0.01 m^3^ min^−1^. The outflow from the drum is introduced into a 19-mm diameter stainless steel tube extending around the perimeter of the exposure room at floor level. The tube has a series of 1100 holes spaced 1 cm apart to insure uniform gas delivery into the room. The generation drum is provided with exhaust valves to vent the radon outdoors when not in use. The radon concentration in the chamber can be controlled over a range of 37 to 3700 Bq m^−3^

#### 6.1.3 Environmental Conditioning System

Two 0.2-m diameter ducts with control dampers are the input/output connections to an externally located environmental conditioning system forming a closed loop with the exposure room. The conditioning system contains a refrigeration unit for temperature control and a steam generation unit for humidity control. Sensors for monitoring temperature and humidity are located in the exposure room and are displayed on the controller panel. Air flow through the system can be varied from 1.9 to 16 m^3^ min^−1^ corresponding to air exchange rates of 0.09 to 0.8 min^−1^ in the exposure chamber. The exposure chamber is maintained at a slight positive pressure relative to the prevailing atmospheric pressure. This eliminates the infiltration of outside room air into the exposure chamber. The temperature and humidity in the chamber can be controlled over the range of 2 to 45 °C and 15% to 100%, respectively.

#### 6.1.4 Continuous Monitoring of the Radon Concentration

The radon concentration in the exposure chamber is monitored continuously from four different locations inside the chamber. Each monitoring system is a 2-L plastic scintillation cell mounted on a 12.5-cm diameter photomultiplier tube. The radon concentration measurements are logged into the data acquisition system at preset time intervals. Typically, hourly concentration results from each system are averaged every 3 h. The radon concentration measurements obtained with the four independent systems are usually within 5% of each other over the entire operating range. The accuracy of the monitors is checked periodically by collecting samples in the exposure chamber and measuring them in the pulse ionization chambers.

## 7. EML Participation in National Intercomparison Exercises

The DOE Office of Health and Environmental Research has funded a number of programs in the radon research area. In an effort to assure the quality of the results obtained from these programs as well as those sponsored by other agencies, EML developed a national radon intercomparison program which is open to any group in the public or private sector which conducts surveys or research programs on the indoor concentration of radon.

EML has conducted 16 radon intercomparison exercises since April 1981. The most recent in a series of reports that describe these exercises summarizes the philosophy and protocol for participation, as well as potential and actual problems in the collection and measurement of radon with various devices [4]. The EML staff has gained experience in the handling and filling of a variety of samplers from over 50 facilities including representation from Canada, the Federal Republic of Germany and Sweden.

At radon concentrations ranging from 220 to 3040 Bq m^−3^ it has been found that 60% to 90% of the participants report results within ± 10% of the value obtained from measurements with the EML pulse ionization chambers. Virtually all participants agree with the nominal EML value within ±25%. These findings serve as an independent check on the validity of EML radon measurements.

## 8. EML Participation in International Intercomparisons

Since 1983, EML has expanded its efforts to include formal participation in an international quality assurance program aimed at the collection and correlation of valid information on the concentration of radon and progeny in the indoor environment assembled for epidemiological and dosimetric risk modeling purposes. Under the auspices of the Nuclear Energy Agency of the Organization for Economic Cooperation and Development (OECD) and the Radiation Protection Programme of the Commission of the European Communities (CEC), the International Intercomparison and Intercalibration Programme for Radon, Thoron, and Daughters Measuring Equipment (HIP) was conceived and became operational in 1983. The principal feature of the HIP is its designation of four laboratories expert in these measurements to act as regional reference centers for quality assurance activities. The four laboratories are: EML (environmental concentrations); the USDI Bureau of Mines, Denver, CO, USA (occupational concentrations); the National Radiological Protection Board, Didcot, Oxfordshire, UK; the Australian Radiation Laboratory, Yallambie, Victoria, Australia. Samplers containing radon from each regional laboratory’s exposure chamber were exchanged and measured at the four laboratories. Of the four laboratories, three perform their radon measurements with scintillation cells, while EML’s primary radon measurements are performed in the fast pulse ionization chambers. Three rounds of sample exchanges were completed over an 18 month period. The results of these international radon intercomparisons have been the subject of two reports [5,6]. At present, radon measurements performed at three of the laboratories agree within the replication error, while the fourth laboratory differs significantly (approximately 9% lower) from the others. The cause of this difference is under investigation and may be resolved through the measurement of a series of special radon samples prepared by NIST.

The IIIP is currently sponsored by the CEC and the International Atomic Energy Agency (IAEA), Vienna, Austria. Its activities are expected to expand in response to the development of the new IAEA-sponsored radon research program. EML will continue to sponsor regional radon and progeny intercomparisons as part of this effort as well as the U.S. Department of Energy’s radon quality assurance program.

## 9. Summary

The EML pulse ionization chambers for radon measurements and the radon calibration room for exposures, while no longer unique, have been instrumental in alerting the scientific community to the needs, requirements, and the subsequent development of appropriate tools for quality assessment. Valuable guidance for the development of a program for the calibration, standardization, and quality assurance for measurements of radon and radon progeny in air has been given by the National Council on Radiation Protection and Measurements [7].

With the present state of technology, no suitable radon gas standard is available to calibrate instruments and assess the quality of measurements produced in measurement programs. In the absence of such a standard, calibration of an instrument must be based on a laboratory standard derived from a NIST SRM ^226^Ra solution. As a practical approach for research and quality assurance purposes, exposure chambers with a fixed radon concentration have been constructed at a number of facilities. This represents a best effort approach to provide some form of quality assurance for a variety of purposes.

## Figures and Tables

**Table 1:** NIST ^226^Ra SRM series, 1940–1984

NIST series	Series units	Calibration instruments	Total uncertainty (%)
1940	μg per 5 mL	Electroscope	?
1947	μg per 5 mL	Electroscope	0.8
1957	ng per 100 mL	Radiation balance (electroscope)	0.3 (1967-changed to 1.5)
1965	μg per 5 mL	Radiation balance 4 *π-γ* ionization chamber	0.5
1967	μg per 5 mL	Radiation balance 4 *π-γ* ionization chamber	3.6
1978	pg per g	4 *π-γ* ionization chamber compared to 1957 SRM	1.34
1984	Pg per g	4 *π-γ* ionization chamber calibrated with national radium standards	1.30

**Table 2:** EML pulse ionization chamber calibrations by the radon emanation method

Year	NIST series	EML calibration factor (cph pg^−1^)
1960	1947	221 (1.8%)
1961	1957	225 (2.2%)
1975	1965	236 (3.0%)
1978	1978	236 (3.0%)
1984	1984	229 (2.2%)
Mean and SD	229 (3.1%)	
Random errors: 3.1% SD from ionization chamber measurements	Systematic errors: 1.30% NIST uncertainty 0.10% Rn half-life 0.44% Ra half-life 0.25% gravimetric measurements	
Estimated Total Uncertainty; 5.2%		
